# Characterization of juice fermented with *Lactobacillus plantarum* EM and its cholesterol‐lowering effects on rats fed a high‐fat and high‐cholesterol diet

**DOI:** 10.1002/fsn3.1217

**Published:** 2019-09-27

**Authors:** Yu Bin Jeon, Jae‐Joon Lee, Hae Choon Chang

**Affiliations:** ^1^ Department of Food and Nutrition Kimchi Research Center Chosun University Gwangju Republic of Korea

**Keywords:** hypercholesterolemic rat, hypocholesterolemic effect, juice fermentation, *Lactobacillus plantarum* EM, starter culture

## Abstract

The aim of this study was to investigate the ability of *Lactobacillus plantarum* EM as a starter culture to control cabbage–apple juice fermentation and to explore the cholesterol‐lowering effects of the fermented juice (EM juice) in rats. *L. plantarum* EM produced strong antimicrobial activities against bacteria and fungi, suppressing other microorganisms in the fermented juice, and was the dominant organism during fermentation and storage. The EM juice also showed strong and broad‐spectrum antimicrobial activity. Rats fed a high‐fat and high‐cholesterol diet and administered EM juice showed significantly reduced total cholesterol (TC), triglyceride, and LDL‐cholesterol levels, as well as a reduced atherogenic index, lower cardiac factors in serum, and lower TC levels in the liver, while total lipid and TC levels in the rat feces increased. Reverse transcription–polymerase chain reaction analysis revealed that the hepatic mRNA expression of HMG‐CoA reductase decreased and the expressions of cholesterol 7α‐hydroxylase and low‐density lipoprotein receptor increased in rats administered EM juice. The effects of EM juice on rats included inhibition of cholesterol synthesis as well as enhancement of cholesterol uptake and cholesterol excretion. The results of this study indicate that the use of *L. plantarum* EM as a functional starter culture for juice fermentation exerts microbial control, enhances sanitary safety, and provides beneficial food effects against hypercholesterolemia.

## 1 INTRODUCTION

The Korean Ministry of Health and Welfare has reported that 11.9%, 15.9%, and 17.6% of Koreans over 30 years of age suffer from diabetes, hypercholesterolemia, and hypertriglyceridemia, respectively (Ministry of Health & Welfare, 2013). Hyperlipidemia, especially hypercholesterolemia, is a major risk factor for the progression and development of atherosclerosis and ischemic heart disease (Berbée et al., 2015; Wu et al., 2013), which are important causes of death worldwide (Cassar, Holmes, Rihal, & Gersh, 2009).

Some lactic acid bacteria (LAB) have been reported to have cholesterol‐lowering effects in animals and humans (Bosch et al., 2014; Jeun et al., 2010; Jo, Choi, Lee, & Chang, 2015; Lee et al., 2010). Moreover, several mechanisms associated with serum cholesterol reduction by LAB have been proposed, including assimilation, surface binding, incorporation into cellular membranes, coprecipitation with deconjugated bile, enzymatic deconjugation of bile acids, conversion of cholesterol, and production of short‐chain fatty acids (Choi & Chang, 2015).

Fermented foods have a long history of consumption worldwide (Marco et al., 2017), and fermentation has traditionally been used as a food preservation method. Moreover, the organoleptic properties of raw materials can be improved during fermentation. Fermented foods can also have enhanced nutritional and functional properties as a result of the formation of bioactive end‐products during fermentation (Marco et al., 2017). It is increasingly considered that some fermented foods have health‐promoting effects that extend well beyond those of the raw food material, and recent human‐based clinical studies of fermented foods support this suggestion (Parvez, Malik, Ah Kang, & Kim, 2006). Accordingly, the market for fermented foods has exhibited remarkable growth over the last few years (Ohm, Kim, & Jun, 2012). The production of fermented foods derived by using a functional starter culture can be expected to provide additional benefits such as improved product shelf‐life and safety, as well as organoleptic, nutritional, or health‐related advantages (Leroy & De Vuyst, 2004).

In our previous study, we isolated *Lactobacillus plantarum* EM from kimchi and observed that growing, resting, and even dead bacterial cells were capable of cholesterol removal via the enzymatic assimilation and cell surface‐binding abilities of *L. plantarum* EM in vitro. Moreover, *L. plantarum* EM appeared to meet the functional criteria required for consideration as a beneficial probiotic, including acid and bile tolerance, antagonistic activity against pathogens, and antibiotic susceptibility (Choi & Chang, 2015).

For further application, we utilized *L. plantarum* EM as a functional starter culture for cabbage–apple juice fermentation. Thereafter, we characterized the fermented juice (EM juice) and evaluated its functionality in rats. The specific goals of this study were to determine the ability of *L. plantarum* EM to control juice fermentation and to investigate the health‐promoting (cholesterol‐lowering) effects of EM juice in high‐fat and high‐cholesterol diet‐induced hypercholesterolemic rats.

## 2 MATERIALS AND METHODS

### 2.1 Cultures and media

LAB strains were cultivated at 30℃ for 24 hr in de Man Rogosa Sharpe (MRS; Difco) medium. *Micrococcus luteus* ATCC 13513 and *Staphylococcus aureus* KCCM 40881 were cultivated in tryptic soy broth (TSB; Difco), while *Vibrio parahaemolyticus* KCCM 11965 was grown in nutrient broth (NB; Difco) supplemented with 2% NaCl. The other bacterial strains used in this study were cultivated in Luria–Bertani (LB; Difco) medium. These strains were incubated at 30–37℃ with shaking for 24 hr (Lee & Chang, 2018).

The fungal strains used in this study were grown on malt extract agar (MEA; Difco) or potato dextrose agar (PDA; Difco) at 30℃ for 24–48 hr, whereas *Penicillium roqueforti* ATCC 10110 were cultivated at 25℃. The ATCC strains were purchased from the American Type Culture Collection (ATCC), whereas the KCCM strains were acquired from the Korean Culture Center of Microorganisms (KCCM). The PF‐2 and PF‐3 fungal strains were isolated by our laboratory (Lee & Chang, 2016).

### 2.2 Preparation of fermented cabbage–apple juice

Cabbage (*Brassica oleracea* var. *capitata*) and apples (*Malus pumila* var*. dulcissima* Koidz) were cleaned under running water and pressed separately by using a juice extractor (Hurom, HD‐RBF09). The two kinds of pressed juice were subsequently blended in equal amounts. *L. plantarum* EM was cultured overnight in MRS broth, then harvested (10,100 *g*, 15 min, 4℃, Hanil Science), resuspended in sterilized distilled water, and inoculated (up to 10^6^ CFU/ml) into the prepared juice, which was then designated as EM juice. The juice without an *L. plantarum* EM inoculum was designated as control juice. The prepared juices were allowed to ferment at 15℃ for 5 days, after which they were stored at 4℃ for 21 days.

### 2.3 Isolation and identification of LAB from the control juice

Microorganisms that formed a clear zone on MRS plates containing 2% (w/v) CaCO_3_ after 24–48 hr culture at 30℃ were isolated from the fermented control juice. The isolated strains were tentatively considered LAB and identified by using morphological observation, Gram‐staining, catalase testing, and determination of 16S rRNA gene sequences (Applied Biosystem). The obtained 16S rRNA gene sequences were subsequently compared with sequences available in the GenBank database (https://blast.ncbi.nlm.nih.gov/Blast.cgi) by using the BLAST program.

### 2.4 Effects of temperature on the growth of LAB

The LAB used in this study were cultivated at 10℃, 15℃, 20℃, and 30℃, respectively, in MRS broth for 120 hr. During cultivation, LAB growth was measured every 24 hr by measuring broth turbidity at 580 nm (Ultrospec 2100 pro; Amersham).

### 2.5 Analysis of juice properties

#### 2.5.1 Acidity and pH assay

Juice pH was measured by using a pH meter (Denver). To determine the titratable total acidity, a juice sample was titrated with 100 mmol/L NaOH to pH 8.3 (Chang & Chang, 2010).

#### 2.5.2 Microbial analysis

Juice samples were serially diluted and spread onto plate count agar (PCA, Merck) to obtain total microbial counts, MRS agar and MRS + 2% CaCO_3_ agar to obtain LAB counts, and 3M™ Petrifilm™ *E. coli*/Coliform Count Plates (3M Corp.) to obtain coliform bacterial counts.

The culture‐independent analysis was conducted simultaneously by performing PCR–DGGE as previously described (Moon, Kim, & Chang, 2018). Briefly, juice samples were centrifuged (13,475 *g*, 5 min, 4℃), after which cell pellets were washed with sterile water and the DNA was extracted by using a Genomic DNA preparation kit (Qiagen). The 16S rRNA gene of the extracted DNA was amplified with the 27F and 1492R 16S universal primers. Thereafter, nested PCR targeting the V3 region of the 16S rRNA gene was conducted with the DGGE primers of *Lac1* and GC *Lac2*. The DGGE was conducted by using 8% (w/v) acrylamide in a Dcode system (BioRad). Bands of interest were extracted from the gels, reamplified using the *Lac1* and GC *Lac2* DGGE primers, and sequenced using an automated DNA sequencer (Applied Biosystem). The determined sequences were then used for BLAST searches of the GenBank database to identify the closest known relatives.

#### 2.5.3 Antimicrobial activity assay

To determine the antimicrobial activity of LAB cultured in MRS or juice, spot‐on‐the‐lawn assays (Lee & Chang, 2016) or paper disk assays (Yang & Chang, 2010) were used. Briefly, LAB strain was cultivated in MRS broth at 30℃ for 24 hr or in juice at 15℃ for 0–5 days. The culture was then centrifuged (10,100 *g*, 15 min, 4℃) and the supernatant sterilized by passing through a 0.45 μm membrane filter (Sartorius) and used as culture filtrate or juice filtrate.

The prepared culture filtrate or juice filtrate was used for the antimicrobial activity assay. Plates were prepared by adding fungi (6.0 log spore per 20 ml MEA or PDA) to 1.5% bacto agar (Duchefa) for antifungal assays or by spreading the bacteria (6.0 log CFU/mL) onto MRS, LB, TSB, or NB + 2% NaCl agar for antibacterial assays (Lee & Chang, 2016; Yang & Chang, 2010). For the paper disk assay, paper disks (diameter 8 mm; Advantec, Tokyo, Japan) on MRS plates were spotted with 100 µl of sample. The plates were then incubated at 30℃ for 24 hr and examined for inhibition zones. For the spot‐on‐the‐lawn assay, 10 µl of sample was spotted onto the sensitive mold and bacterial plates. Antimicrobial activity, which was defined as the reciprocal of the highest dilution at which microbial growth was inhibited, was expressed as arbitrary units (AU) per milliliter.

### 2.6 Animals and diets

To investigate the cholesterol‐lowering effects of the juice, 5‐week‐old Sprague Dawley male rats (*n* = 32) were purchased from Orient Bio Inc. (Daejeon, Korea). The rats were housed in stainless steel cages under controlled temperature (22 ± 1°C) and humidity (60%) conditions with a 12‐hr light–dark cycle and were fed a diet of commercial pelleted rat chow (Samtako) for 7 days. Animals were then randomly allocated into one of the following four groups (*n* = 8): (a) ND, normal diet; (b) HFCD, high‐fat/high‐cholesterol diet; (c) HFCD‐A, high‐fat/high‐cholesterol diet + nonfermented control juice; and (d) HFCD‐B, high‐fat/high‐cholesterol diet + fermented EM juice. Juice was orally administrated to each rat at a volume of 1 ml/day, and the same amount of distilled water was administrated to rats in the ND and HFCD groups. The feed of the ND group consisted of AIN‐93 purified diet containing 17.48% protein, 6.85% fat, 62.99% carbohydrates, 4.08% ash, 1.48% minerals, and 0.68% vitamins (Reeves, Nielsen, & Fahey, 1993). The HFCD consisted of 17% fat and 1% cholesterol (w/w) excluding the AIN‐93 purified diet. Rat food intake and body weight were recorded daily and weekly, respectively, during the 6‐week feeding period. All animal procedures were performed in accordance with the Guidelines for Institutional Animal Care and Use Committee of Chosun University, Korea (CIACUC2016‐A0032).

### 2.7 Analysis of lipid profiles in animals

#### 2.7.1 Analysis of biochemical parameters

After overnight fasting, final body weight was measured and rats were sacrificed. Blood samples were then collected by cardiac puncture and used to determine serum lipid profiles. The liver was immediately removed, rinsed with 0.9% saline solution, weighed, and flash‐frozen for further analysis. Feces were collected for 5 consecutive days before euthanasia, completely dried by heating at 70°C for 3 days, and stored at −70°C until lipid profile analyses were performed. Serum levels of triglyceride (TG), total cholesterol (TC), and high‐density lipoprotein (HDL) cholesterol were determined by using an auto blood analyzer (Fuji Dri‐Chem 3500, Fujifilm, Tokyo, Japan). Serum low‐density lipoprotein (LDL) cholesterol was calculated according to the Friedewald method (Friedewald, Levy, & Fredrickson, 1972). Both the atherogenic index (AI) and the cardiac risk factor (CRF) were calculated using the following formulae: AI = (TC–HDL‐cholesterol)/HDL‐cholesterol and CRF = TC/HDL‐cholesterol (Rosenfeld, 1989). Lipids were extracted from the livers and feces by using the Folch method (Folch, Lees, & Sloane Stanley, 1957), while TG and TC contents in the liver and feces were measured as described by Rizvi et al. (2003).

#### 2.7.2 Hepatic RNA extraction and reverse transcription–polymerase chain reaction (RT‐PCR)

Total RNA was extracted from frozen rat livers by using an RNeasy mini kit (Qiagen, Gaithersburg, MD, USA). Complementary DNA (cDNA) was synthesized by using SuperScript III Reverse Transcriptase and oligo‐dT primers (Invitrogen) according to the manufacturer's protocols. PCR (TaKaRa Biochemicals) analysis of the cDNA gene expression was conducted by using the following PCR primers: forward 5'‐GTGATTACCCTGAGCTTAGC‐3' and reverse 5'‐TGGGATGTGCTTAGCATTGA‐3′ for 3‐hydroxy‐3‐methylglutaryl‐CoA reductase (HMGCR; Alvarez, Gómez, Scardapane, Fornes, & Giménez, 2007); forward 5′‐GCCGTCCAAGAAATCAAGCAGT‐3′ and reverse 5′‐TGTGGGCAGCGAGAACAAAGT‐3′ for cholesterol 7α‐hydroxylase (CYP7A1; Sun, Xie, Xue, & Wang, 2010); forward 5′‐ATTTTGGAGGATGAGAAGCAG‐3′ and reverse 5′‐CAGGGCGGGGAGGTGTGAGAA‐3′ for low‐density lipoprotein receptor (LDLR; Nakamura et al., 2009); forward 5′‐GTGGGGCGCCCCAGGCACCAGGGC‐3′ and reverse 5′‐CTCCTTAATGTCACGCACGATTTC‐3′ for β‐actin (Jang et al., 2003). The PCR conditions were as follows: 30 cycles of 94°C for 3 min, 60°C for 1 min, and 72°C for 2 min for HMGCR, CYP7A1, and LDLR; 30 cycles of 95°C for 15 s, 60°C for 60 s, and 72°C for 30 s for β‐actin. The relative amount of each studied mRNA was normalized to β‐actin as a housekeeping gene, and the data were analyzed by using Alpha Ease FC software (Alpha Innotech Corporation).

### 2.8 Statistical analysis

Data pertaining to the chemical components analysis and sensory evaluations were expressed as means ± standard deviations (*SD*) of duplicate evaluations, and comparisons of means were made by using the *t* test and Duncan's multiple range test, respectively. Animal experimental data were expressed as means ± standard errors (SE) and were analyzed by performing one‐way analysis of variance followed by Tukey's posthoc test by using the SPSS ver. 11.5 software (SPSS). A *p* < .05 was considered to indicate statistical significance.

## 3 RESULTS AND DISCUSSION

### 3.1 Screening of microorganisms in cabbage–apple juice (control juice)

To identify the microorganisms naturally present in the prepared cabbage–apple juice, screening of microorganisms in the control juice was conducted. Total microorganisms and LAB in the control juice were enumerated, and their morphologies were evaluated on plates (PCA, MRS, and MRS + 2% CaCO_3_) after cultivation at 30℃ for 24–48 hr. When the control juice was freshly prepared, 4.18 log CFU/ml of viable cells composed of different bacteria was detected. However, 9.03 log CFU/ml of LAB were detected among 9.08 log CFU/ml of total viable cells after 5 days of fermentation at 15℃. The counted LAB were divided into three strains (HL1, HL2, and HL3) based on colony color and cell‐shape (data not shown). All three selected strains were Gram‐positive and catalase‐negative. When 16S rRNA gene sequences of the three strains, HL1 (1377 bp), HL2 (1482 bp), and HL3 (1443 bp), were determined and compared with those of type strains in GenBank, sequences of strain HL1 were found to be 99.8% homologous with those of *Weissella cibaria* Ⅱ‐Ⅰ‐59^T^, while sequences of strains HL2 and HL3 showed 99.9% and 99.4% homologies with the 16S rRNA gene sequences of *Leuconostoc mesenteroides* ATCC 8293^T^. Thus, HL1, HL2, and HL3 were designated as *W. cibaria* HL1, *Leu. mesenteroides* HL2, and *Leu. mesenteroides* HL3, respectively, and their sequences were deposited in GenBank (accession number KY229730 for *W. cibaria* HL1, KY233187 for *Leu. mesenteroides* HL2, and KY233186 for *Leu. mesenteroides* HL3).

### 3.2 Characteristics of LAB

**Figure 1:**
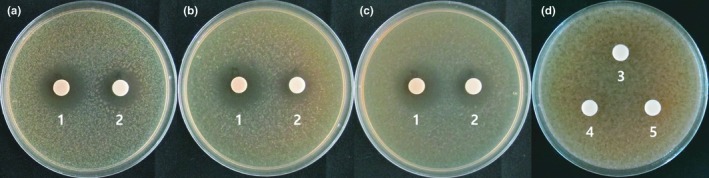
Antimicrobial activity of lactic acid bacteria. *Lactobacillus plantarum* EM (1), *Weissella cibaria* HL1 (3), *Leuconostoc mesenteroides* HL2 (4), or *Leu. mesenteroides* HL3 (5) was cultivated in MRS for 1 day at 30℃. Simultaneously, *L. plantarum* EM (2) was cultivated in prepared cabbage–apple juice for 5 days at 15℃. Their activities were then assayed against *W. cibaria* HL1 (a), *Leu. mesenteroides* HL2 (b), *Leu. mesenteroides* HL3 (c), and *L. plantarum* EM (d) by performing paper disk assays

We applied *L. plantarum* EM as a starter culture for the cabbage–apple juice in an attempt to control fermentation. However, juice fermentation also occurred in response to fermentation with *W. cibaria* HL1, *Leu. mesenteroides* HL2, and *Leu. mesenteroides* HL3; thus, we attempted to determine the level of antimicrobial activity of *L. plantarum* EM against the three predominant microorganisms in the juice. *L. plantarum* EM cultivated in MRS or juice showed strong antimicrobial activity against *W. cibaria* HL1, *Leu. mesenteroides* HL2, and *Leu. mesenteroides* HL3. However, *W. cibaria* HL1, *Leu. mesenteroides* HL2, and *Leu. mesenteroides* HL3 did not show any antimicrobial activities against *L. plantarum* EM (Figure 1). These results imply that the use of *L. plantarum* EM as a starter culture can suppress the three main microorganisms originally present during juice fermentation.

**Figure 2:**
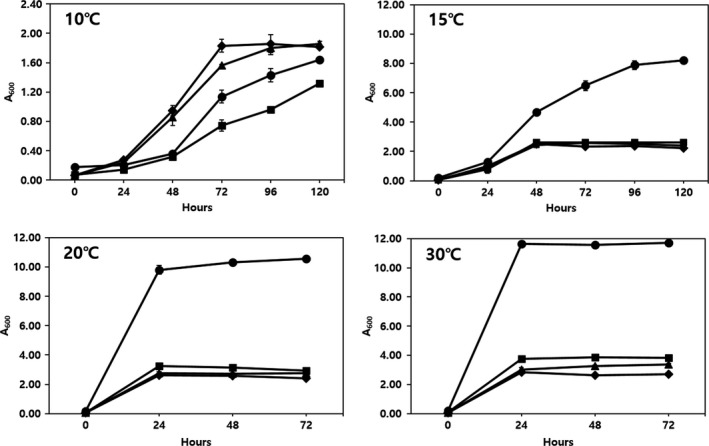
Effects of temperature on the growth of lactic acid bacteria. *Lactobacillus plantarum* EM (●),*Weissella cibaria* HL1 (■),*Leuconostoc mesenteroides* HL2 (◆), and *Leu. mesenteroides* HL3 (▲)

We also tried to determine the effects of temperature on the growth of *L. plantarum* EM, *W. cibaria* HL1, and *Leu. mesenteroides* HL2 and *Leu. mesenteroides* HL3 (Figure 2). *W. cibaria* HL1 showed the least growth at 10℃, followed by *L. plantarum* EM, whereas *Leu. mesenteroides* HL2 and HL3 grew better than *L. plantarum* EM at 10℃. However, the growth of *L. plantarum* EM was notably higher (approximately 3–4 times) than that of the other three LAB strains at 15℃, 20℃, and 30℃. It is important to establish the lowest fermentation temperature possible to maintain the freshness of the juice; therefore, we decided that the optimum temperature for juice fermentation using *L. plantarum* EM was 15℃ in order to minimize the growth of the innate LAB strains as well as to maintain juice freshness (Figure 2).

### 3.3 Characteristics of the juice

#### 3.3.1 Acidity, pH, and viable cell counts

When the juice was initially prepared, its total acidity was approximately 0.21% (pH 5.52). During fermentation at 15℃ for 5 days and then storage at 4℃ for 21 days, the acidity of the juice fermented using *L. plantarum* EM (EM juice) had increased faster than that of the control juice. Specifically, the acidity of the EM and control juices reached 1.12% (pH 3.91) and 0.89% (pH 4.14) at day 5. The acidities of both juices increased continuously, reaching 1.46% (pH 3.73) and 1.19% (pH 4.12), respectively, after 7 days of storage. Thereafter, the acidities increased slowly, reaching 1.58% (pH 3.69) for EM juice and 1.26% (pH 4.08) for control juice at day 21 of storage (Table 1).

**Table 1:** Changes in pH, acidity, and microbial populations in juice

Juice	Characteristics		Fermentation (days) at 15℃						Storage (days) at 4℃		
			0	1	2	3	4	5	7	14	21
EM juice	pH		5.52 ± 0.06^a^	5.39 ± 0.07^a^	4.72 ± 0.06^b^	4.27 ± 0.11^c^	4.03 ± 0.10^d^	3.91 ± 0.07^d^	3.73 ± 0.01^e^	3.69 ± 0.01^e^	3.68 ± 0.03^e^
	Acidity (%)		0.21 ± 0.02^e^	0.26 ± 0.03^e^	0.54 ± 0.06^d^	0.79 ± 0.07^c^	1.01 ± 0.07^b^	1.12 ± 0.06^b^	1.46 ± 0.04^a^	1.54 ± 0.02^a^	1.58 ± 0.04^a^
	Microbial	Total viable cells	6.74 ± 0.03^d^	7.84 ± 0.12^c^	8.82 ± 0.10^b^	9.03 ± 0.06^a^	9.09 ± 0.01^a^	9.11 ± 0.03^a^	9.09 ± 0.03^a^	9.06 ± 0.12^a^	9.01 ± 0.01^a^
	Population	Lactic acid bacteria	6.58 ± 0.07^e^	7.79 ± 0.03^d^	8.93 ± 0.06^c^	9.09 ± 0.07^ab^	9.09 ± 0.07^ab^	9.10 ± 0.06^ab^	9.09 ± 0.01^ab^	9.15 ± 0.02^a^	9.00 ± 0.03^b^
	(log CFU/mL)	Dominance of EM, %	100.0	100.0	100.0	100.0	100.0	100.0	100.0	100.0	100.0
Control juice	pH		5.56 ± 0.03^a^	5.55 ± 0.01^a^	5.05 ± 0.04^b^	4.62 ± 0.04^c^	4.23 ± 0.03^d^	4.14 ± 0.06^e^	4.12 ± 0.01^e^	4.08 ± 0.01^e^	4.08 ± 0.02^e^
	Acidity (%)		0.22 ± 0.01^g^	0.23 ± 0.01^g^	0.38 ± 0.04^f^	0.53 ± 0.05^e^	0.76 ± 0.02^d^	0.89 ± 0.04^c^	1.19 ± 0.01^b^	1.24 ± 0.02^ab^	1.26 ± 0.04^a^
	Microbial	Total viable cells	4.09 ± 0.12^f^	4.74 ± 0.06^e^	7.28 ± 0.03^c^	8.06 ± 0.08^b^	9.13 ± 0.02^a^	9.18 ± 0.14^a^	8.97 ± 0.10^a^	8.07 ± 0.10^b^	6.05 ± 0.09^d^
	Population	Lactic acid bacteria	3.42 ± 0.01^g^	4.69 ± 0.07^f^	7.23 ± 0.06^d^	8.02 ± 0.02^c^	9.13 ± 0.04^a^	9.15 ± 0.15^a^	8.96 ± 0.05^b^	8.08 ± 0.03^c^	6.09 ± 0.06^e^
	(log CFU/mL)	(short rod/coccus, %)	(62.3/37.7)	(58.9/41.1)	(51.3/48.7)	(50.5/49.5)	(50.0/50.0)	(47.9/52.1)	(50.6/49.4)	(65.2/34.8)	(66.9/33.1)

Note

Values are the means ± *SD* from triplicate determination. Means with different superscripts in the same row indicate significant differences (*p* < .05) by Duncan's multiple range test.

As shown in Table 1, the initial viable counts of total cells and LAB were 6.74 log CFU/ml and 6.58 log CFU/ml for EM juice and 4.09 log CFU/ml and 3.42 log CFU/ml for control juice, respectively. The higher initial cell counts in EM juice were due to inoculation with the starter culture. Following fermentation at 15℃, maximum cell growth (9.01–9.11 log CFU/ml) was obtained from day 3 of fermentation, and these levels were maintained until day 21 of storage. All LAB cells detected during the fermentation and storage were *L. plantarum* EM. However, in the control juice, the initial cell counts increased to maximum values of 9.18 log CFU/ml of total cells and 9.15 log CFU/mL of LAB cells after 5 days of fermentation. Counts of LAB from control juice rapidly decreased during storage to 6.09 CFU/ml at day 21 of storage. The ratio of short rod‐type:coccus‐type LAB was 62.3:37.7 at day 0 of fermentation, after which the proportion of short rod‐type LAB decreased until day 5 of fermentation, then increased from day 7 of storage until reaching a final ratio of 66.9:33.1 at day 21 of storage. These results indicate that the short rod‐type LAB (possibly *W. cibaria* HL1) in the juice was more stable than the coccus‐type LAB (possibly *Leu. mesenteroides* HL2 and HL3) during storage. No coliform bacteria were detected in the EM or control juices during fermentation and storage.

**Figure 3:**
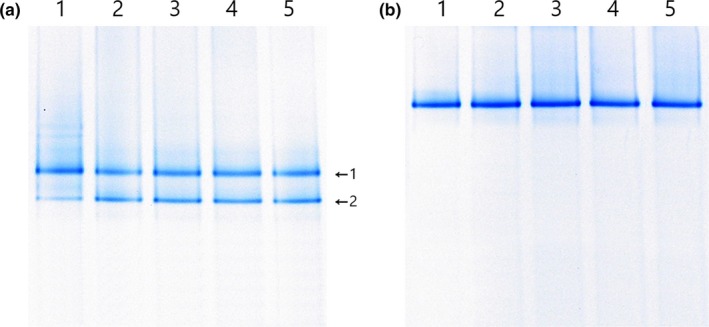
PCR–DGGE patterns of 16S V3 rRNA gene sequences. PCR–DGGE patterns of 16S V3 rRNA gene sequences in control juice (non‐*Lactobacillus plantarum* EM‐fermented juice) (a) and EM juice (*L. plantarum* EM‐fermented juice) (b). 1:0 days of fermentation, 2:5 days of fermentation, 3:7 days of storage, 4:14 days of storage, and 5:21 days of storage. The closest relatives of the fragments were identified and compared using sequences from GenBank

The microbial populations of juice were analyzed simultaneously by performing PCR–DGGE (Figure 3). The results were similar to the plate count results. In the control juice (Figure 3a) at day 0 of fermentation, two bands were identified as *W. cibaria* (band 1) and *Leu. mesenteroides* (band 2) with 99% identities. The same two bands were continually detected as thick bands until day 21 of storage. In the EM juice, only one band, identified as *L. plantarum* EM (with 100% identity), was detected during fermentation and storage (Figure 3b). The results demonstrate that *L. plantarum* EM suppressed the innate microorganisms in the juice and controlled juice fermentation efficiently.

#### 3.3.2 Antimicrobial activity of juice

After 5 days of juice fermentation at 15℃, the antimicrobial activity was investigated (Table 2). *L. plantarum* EM cultivated in MRS at 30℃, which was used as a control, showed the highest antimicrobial activities against the tested pathogenic bacteria and food spoilage fungi. The EM juice also showed strong antifungal (40–160 units) and antibacterial (0–2560 units) activities, even though its activities were somewhat lower than those of *L. plantarum* EM cultured in MRS at 15℃. However, the activities of EM juice against different microorganisms were significantly higher than those of the control juice (Table 2). Such antimicrobial activities of EM juice might result in improved sanitary safety along with extended shelf‐life of the juice.

**Table 2:** Antimicrobial activity of *Lactobacillus plantarum* EM and fermented juices

Micro organism	Sensitive strain	Sample	Unit (AU/mL)					
			Culture/Fermentation period (days)*					
			0	1	2	3	4	5
Gram‐positive bacteria	*Bacillus cereus* ATCC 14579	EM/MRS at 30℃	0	320	320	320	320	320
		EM/MRS at 15℃	0	0	40	80	160	160
		Control juice	0	0	0	0	0	20
		EM juice	0	0	20	40	80	160
	*Micrococcus luteus* ATCC 13513	EM/MRS at 30℃	0	40	40	40	40	40
		EM/MRS at 15℃	0	0	0	0	20	40
		Control juice	0	0	0	0	0	0
		EM juice	0	0	0	0	0	0
	*Staphylococcus aureus* KCCM 40881	EM/MRS at 30℃	0	40	40	40	40	40
		EM/MRS at 15℃	0	0	0	20	40	40
		Control juice	0	0	0	0	0	0
		EM juice	0	0	0	0	0	20
Gram‐negative bacteria	*Escherichia coli* O157:H7 ATCC 43895	EM/MRS at 30℃	0	160	160	160	160	160
		EM/MRS at 15℃	0	0	20	40	40	80
		Control juice	0	0	0	0	0	0
		EM juice	0	0	0	0	20	40
	*Pseudomonas aeruginosa* KCCM 11328	EM/MRS at 30℃	0	320	320	320	320	320
		EM/MRS at 15℃	0	0	20	80	160	320
		Control juice	0	0	0	0	0	0
		EM juice	0	0	20	40	80	160
	*Salmonella enterica* serovar. Typhi ATCC 14028	EM/MRS at 30℃	0	160	160	160	160	160
		EM/MRS at 15℃	0	0	20	40	80	80
		Control juice	0	0	0	0	0	0
		EM juice	0	0	0	0	20	40
	*Vibrio parahaemolyticus* KCCM 11965	EM/MRS at 30℃	0	5,120	5,120	5,120	5,120	5,120
		EM/MRS at 15℃	0	0	640	1,280	2,560	2,560
		Control juice	0	0	0	0	0	0
		EM juice	0	0	160	640	1,280	2,560
Fungi	*Aspergillus flavus* ATCC 22546	EM/MRS at 30℃	0	320	320	320	320	320
		EM/MRS at 15℃	0	0	40	80	80	160
		Control juice	0	0	0	0	0	20
		EM juice	0	0	40	40	80	80
	*Aspergillus fumigatus* ATCC 96918	EM/MRS at 30℃	0	640	640	640	640	640
		EM/MRS at 15℃	0	0	160	320	320	320
		Control juice	0	0	0	0	0	40
		EM juice	0	0	80	80	80	160
	*Aspergillus ochraceus* PF−2	EM/MRS at 30℃	0	80	80	80	80	80
		EM/MRS at 15℃	0	0	20	40	80	80
		Control juice	0	0	0	0	0	20
		EM juice	0	0	20	20	40	40
	*Penicillium roqueforti* ATCC 10110	EM/MRS at 30℃	0	40	40	40	40	40
		EM/MRS at 15℃	0	0	20	40	40	40
		Control juice	0	0	0	0	0	0
		EM juice	0	0	20	20	20	40
	*Aspergillus nidulans* PF−3	EM/MRS at 30℃	0	320	320	320	320	320
		EM/MRS at 15℃	0	0	40	80	160	320
		Control juice	0	0	0	0	0	20
		EM juice	0	0	40	40	80	160

Note

EM/MRS, *Lactobacillus plantarum* EM was cultivated in MRS broth at 15℃ or 30℃ for 5 days. Control and EM juices were fermented at 15℃ for 5 days.

* Antimicrobial activity was measured by performing spot‐on‐the‐lawn assay (AU/mL) after 0–5 days of cultivation of the sample as described in materials and methods.

### 3.4 Cholesterol‐lowering effects of EM juice in rats fed a high‐fat/high‐cholesterol diet

All rats generally appeared healthy throughout the feeding period. As shown in Table 3, rats belonging to the HFCD group showed significantly higher average body weight gain (*p* < .05) than those of rats in the other groups. The other three groups (ND, HFCD‐A, and HFCD‐B) showed similar body weight gains. There was no significant difference (*p* > .05) in total food intake among the groups.

**Table 3:** Lipid profiles of serum, liver, and fecal samples from rats different diets

Diet*	ND	HFCD	HFCD‐A	HFCD‐B
Body weight gain (g/day)	5.93 ± 0.42^b^	7.13 ± 0.75^a^	6.18 ± 0.59^b^	6.01 ± 0.71^b^
Serum (mg/dL)				
Triglyceride	64.38 ± 5.24^b^	97.50 ± 7.80^a^	88.00 ± 13.09^a^	71.13 ± 9.13^b^
Total cholesterol	83.13 ± 5.00^c^	115.25 ± 11.57^a^	97.75 ± 5.50^b^	84.13 ± 6.42^c^
LDL‐cholesterol	35.13 ± 5.92^c^	75.00 ± 11.35^a^	57.28 ± 7.50^b^	41.90 ± 9.55^c^
HDL‐cholesterol	35.13 ± 3.40^a^	20.75 ± 5.95^c^	22.88 ± 2.64^bc^	28.00 ± 7.09^b^
AI	1.37 ± 0.09^d^	4.55 ± 0.31^a^	3.28 ± 0.29^b^	2.00 ± 0.18^c^
CRF	2.37 ± 0.21^d^	5.55 ± 0.39^a^	4.27 ± 0.28^b^	3.00 ± 0.27^c^
Liver (mg/g)				
Total lipid	118.79 ± 17.27^c^	388.77 ± 51.63^a^	331.70 ± 59.49^b^	318.31 ± 31.63^b^
Triglyceride	85.27 ± 12.73^c^	193.08 ± 21.61^a^	160.36 ± 18.53^b^	151.15 ± 19.78^b^
Total cholesterol	20.22 ± 5.47^d^	58.10 ± 6.95^a^	42.12 ± 6.66^b^	32.59 ± 4.77^c^
Fecal (mg/g)				
Total lipid	127.87 ± 22.14^c^	154.55 ± 22.73^b^	158.70 ± 16.32^b^	170.37 ± 22.32^a^
Triglyceride	33.64 ± 4.89^c^	65.80 ± 5.78^b^	69.76 ± 4.35^ab^	72.25 ± 8.48^a^
Total cholesterol	14.52 ± 2.61^c^	25.26 ± 4.05^b^	24.54 ± 3.91^b^	30.74 ± 4.13^a^

Note

AI (atherogenic index) = (total cholesterol – HDL‐cholesterol)/HDL‐cholesterol. CRF (cardiac risk factor) = total cholesterol/HDL‐cholesterol. Values are the means ± SE of eight rats per group. Values with different superscripts in the same row indicate significant differences (*p* < .05) based on Tukey's test.

* ND, normal diet; HFCD, high‐fat/high‐cholesterol diet; HFCD‐A, high‐fat/high‐cholesterol diet containing control juice (nonfermented juice) and HFCD‐B, high‐fat/high‐cholesterol diet containing EM juice (*Lactobacillus plantarum* EM‐fermented juice at 15℃ for 5 days).

The HFCD group exhibited significantly higher levels of serum TC and LDL‐cholesterol than the other diet groups. Specifically, the HFDC‐B group showed greatly reduced serum TG, TC, and LDL‐cholesterol levels, as well as an increased HDL‐cholesterol level compared with the HFCD group. Among the juice‐treated groups, the HFCD‐B group showed significantly reduced levels of serum TG, TC, and LDL‐cholesterol compared with those in the HFCD‐A group. The AI and CRF values decreased in the order HFCD > HFCD‐A > HFCD‐B > ND.

The hepatic total lipid (TL), TG, and TC contents of the HFCD group were significantly higher than those of the other diet groups. The increased contents of TL, TG, and TC in the HFCD group were significantly lower in the HFCD‐A and HFCD‐B groups. The hepatic TC contents in the HFCD‐A and HFCD‐B groups were reduced by 27.58% and 43.91%, respectively, when compared to the level in the HFCD group.

The amounts of feces in the collected stool samples were similar among the four groups. The highest levels of TL, TG, and TC in fecal samples were found in the HFCD‐B group. When compared with the HFCD group, the TL, TG, and TC levels in the feces of the HFCD‐B group were significantly elevated by 10.24%, 9.80%, and 21.69%, respectively. The contents of fecal TL, TG, and TC were not significantly different between the HFCD and HFCD‐A groups.

Hypercholesterolemia is known to be a major contributor to the development of coronary heart disease (CHD) and fatty liver disease, which results in health problems and death (Harb, Bustanji, & Abdalla, 2018). High‐fat and high‐cholesterol diets are associated with the development of chronic disorders such as CHD, nonalcoholic fatty liver disease, hyperlipidemia, diabetes, obesity, and metabolic syndrome (Harb et al., 2018; Jung & Kim, 2013). In the present study, an obvious increase in serum and hepatic TG and TC, serum LDL‐cholesterol, AI, and CRF, and a decrease in serum HDL‐cholesterol level were observed in hypercholesterolemic rats. However, rats treated with EM juice had effectively lower serum TG, TC, and LDL‐cholesterol levels with no marked decrease in serum HDL‐cholesterol levels. These results indicated that administration of EM juice could ameliorate hypercholesterolemia caused by a high‐fat/high‐cholesterol diet. Additionally, the excretions of TL, TG, and TC via feces following consumption of EM juice, which harbors more than 1.33 × 10^9^ *L. plantarum* EM cells/mL, were significantly higher than those in the other groups.

**Figure 4:**
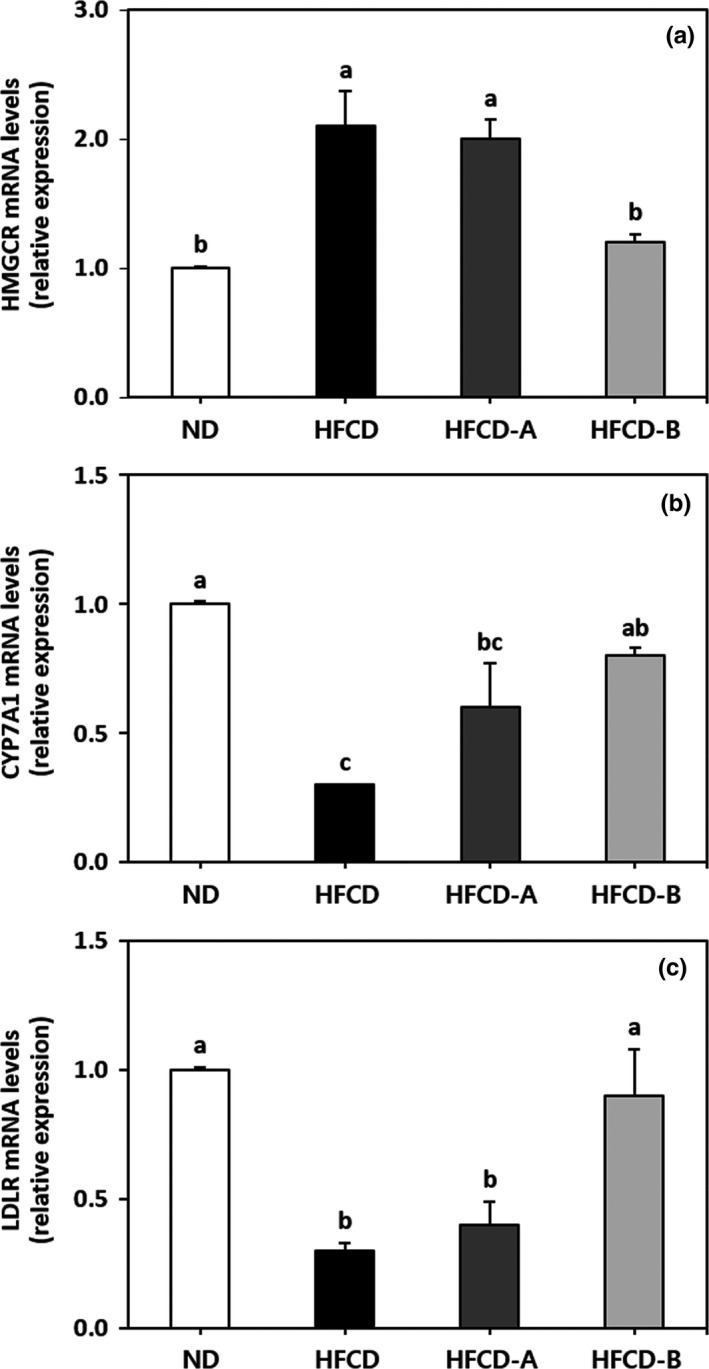
Effects of *Lactobacillus plantarum* EM‐fermented juice on the hepatic mRNA expression of genes in rats. 3‐hydroxy‐3methylglutaryl‐CoA reductase (HMGCR) (a), cholesterol 7α‐hydroxylase (CYP7A1) (b), and lipoprotein receptor (LDLR) (c). Different letters above the bars indicate significant differences (*p* < .05) based on Tukey's test. ND, normal diet; HFCD, high‐fat/high‐cholesterol diet; HFCD‐A, high‐fat/high‐cholesterol diet containing nonfermented control juice and HFCD‐B, high‐fat/high‐cholesterol diet containing fermented EM juice

The mRNA expression levels of hepatic genes HMGCR, CYP7A1, and LDLR are shown in Figure 4. The HFCD group had higher levels of HMGCR mRNA expression, as well as lower levels of CYP7A1 and LDLR mRNA expression than those in the other diet groups. Specifically, the HFCD‐B group showed significantly reduced mRNA expression of HMGCR, as well as increased mRNA expression of CYP7A1 and LDLR when compared with the HFCD group. The mRNA expression levels of all investigated genes did not differ significantly between the HFCD and HFCD‐A group (*p* > .05).

The liver is generally considered to be the primary organ responsible for maintaining cholesterol homeostasis, which occurs via regulation of cholesterol biosynthesis and uptake as well as cholesterol excretion into bile acids (Murakami et al., 2016). The key genes involved in the synthesis, absorption, and degradation of hepatic cholesterol are mainly associated with HMGCR, LDLR, and CYP7A1, respectively (Han et al., 2005). HMGCR is the rate‐limiting enzyme associated with hepatic cholesterol biosynthesis, and a low hepatic HMGCR gene expression level is associated with a lower risk of atherosclerotic lesion formation (Bocan et al., 1998). The LDLR gene mediates the absorption of plasma LDL‐cholesterol into the liver. Therefore, high levels of hepatic LDLR mRNA are associated with improved clearance of plasma LDL‐cholesterol (Brown & Goldstein, 1986). CYP7A1 is the initial and rate‐limiting enzyme involved in the pathway of bile acids biosynthesis from cholesterol in the liver for excretion into bile (Cheema, Cikaluk, & Agellon, 1997). The overexpression of CYP7A1 inhibits the development of atherosclerosis in C57BL/6J mice and prevents diet‐induced hypercholesterolemia (Miyake et al., 2002). It was previously reported that chronic feeding of a high‐fat and/or high‐cholesterol diet led to the suppression of hepatic LDLR and CYP7A1 expression, as well as the promotion of HMGCR expression, which is associated with the development of severe hepatic cholesterol accumulation (Choi, Gwon, Ahn, Jung, & Ha, 2013; Han et al., 2005). In the present study, treatment with HFCD‐B significantly reversed the upregulating effects of HFCD on the expression of HMGCR mRNA and the downregulating effects of HFCD on the expression of CYP7A1 and LDLR mRNA. The decrease in hepatic TC content coincided with the inhibition of HMGCR mRNA expression, as well as with increases in CYP7A1 and LDLR gene expressions in rats fed HFCD‐B when compared with the HFCD group. Consumption of the EM juice was effective at improving serum and hepatic lipid profiles in rats fed a high‐fat/high‐cholesterol diet.

Fermented and nonfermented juices are readily available. As fermented juice with probiotics has been shown to exhibit nutritional and health‐promoting effects (Marazza, LeBlanc, Giori, & Garro, 2013), it is necessary to elucidate the mechanism of those beneficial effects. Some studies have demonstrated that the hypocholesterolemic and hypolipidemic effects of fermented fruit and/or vegetable juices (Jeon, Kim, Mun, Cha, & Yu, 2017; Li et al., 2014) might be associated with the organic acids (Parvez et al., 2006) and short‐chain fatty acids (Li et al., 2014) produced by LAB or with thepolyphenolic compounds derived from juice materials (Hertog et al., 1993; Yugarani, Tan, & T, M., & Das, N. P., 1992) that have antiatherogenic activity.

On the other hand, the cholesterol removal effects of *L. plantarum* EM in this study were observed to occur as a result of its enzymatic assimilation, including bile salt hydrolase activity, and the high cholesterol‐binding capacity of its cell wall (Choi & Chang, 2015). Such cholesterol removal mechanisms of *L. plantarum* EM could be attributed to excessive excretion of the high levels of cholesterol and fat added by diet via the feces as well as to decreases in the serum and liver lipid profiles. Administration of EM juice to hypercholesterolemic rats consequently improved the serum and liver lipid profiles. The results of our study imply that the hypocholesterolemic effects of EM juice are partially mediated through the regulation of hepatic genes involved in cholesterol metabolism and partially connected with a physical interaction between cholesterol molecules and *L. plantarum* EM cell walls. Such effects of the *L. plantarum* EM‐fermented juice on rats resulted in the inhibition of cholesterol synthesis as well as enhancement of cholesterol uptake and a high level of cholesterol excretion.

## 4 CONCLUSION

The *L. plantarum* EM‐fermented juice showed significant hypocholesterolemic effects and cholesterol metabolism improvement in rats. Overall, the results indicate that the use of *L. plantarum* EM as a functional starter culture for juice fermentation can exert microbial control, improve sanitary safety, and provide beneficial food effects in prevention of hypercholesterolemia.

## CONFLICT OF INTEREST

The authors declare that they do not have any conflict of interests.

## ETHICAL APPROVAL

There is no conflict of interest in this study. The study has conformed to the Declaration of Helsinki, US. All experimental protocols, procedures, and animals were reviewed and approved by Institutional Animal Care and Use Committee of Chosun University, Korea (CIACUC2016‐A0032).

## References

[fsn31217-bib-0001] Alvarez, S. M. , Gómez, N. N. , Scardapane, L. , Fornes, M. W. , & Giménez, M. S. (2007). Effects of chronic exposure to cadmium on prostate lipids and morphology. BioMetals, 20, 727 10.1007/s10534-006-9036-9 17066326

[fsn31217-bib-0002] Berbée, J. F. P. , Boon, M. R. , Khedoe, P. P. S. J. , Bartelt, A. , Schlein, C. , Worthmann, A. , … Rensen, P. C. N. (2015). Brown fat activation reduces hypercholesterolaemia and protects from atherosclerosis development. Nature Communications, 6, 6356 10.1038/ncomms7356 PMC4366535 25754609

[fsn31217-bib-0003] Bocan, T. M. , Bak Mueller, S. , Quenby Brown, E. , Lee, P. , Bocan, M. J. , Rea, T. , & Pape, M. E. (1998). HMG‐CoA reductase and ACAT inhibitors act synergistically to lower plasma cholesterol and limit atherosclerotic lesion development in the cholesterol‐fed rabbit. Atherosclerosis, 139, 21–30. 10.1016/s0021-9150(98)00046-x 9699888

[fsn31217-bib-0004] Bosch, M. , Fuentes, M. C. , Audivert, S. , Bonachera, M. A. , Peiró, S. , & Cuñé, J. (2014). *Lactobacillus plantarum* CECT 7527, 7528 and 7529: Probiotic candidates to reduce cholesterol levels. Journal of the Science of Food and Agriculture, 94, 803–809. 10.1002/jsfa.6467 24186773

[fsn31217-bib-0005] Brown, M. S. , & Goldstein, J. L. (1986). A receptor‐mediated pathway for cholesterol homeostasis. Science, 232, 34–47. 10.1126/science.3513311 3513311

[fsn31217-bib-0006] Cassar, A. , Holmes, D. R. , Rihal, C. S. , & Gersh, B. J. (2009). Chronic coronary artery disease: Diagnosis and management. Mayo Clinic Proceedings, 84, 1130–1146. 10.4065/mcp.2009.0391 19955250 PMC2787400

[fsn31217-bib-0007] Chang, J. Y. , & Chang, H. C. (2010). Improvements in the quality and shelf life of kimchi by fermentation with the induced bacteriocin‐producing strain, *Leuconostoc citreum* GJ7 as a starter. Journal of Food Science, 75, M103–M110. 10.1111/j.1750-3841.2009.01486.x 20492238

[fsn31217-bib-0008] Cheema, S. K. , Cikaluk, D. , & Agellon, L. B. (1997). Dietary fats modulate the regulatory potential of dietary cholesterol on cholesterol 7 alpha‐hydroxylase gene expression. Journal of Lipid Research, 38, 315–323. 9162751

[fsn31217-bib-0009] Choi, E. A. , & Chang, H. C. (2015). Cholesterol‐lowering effects of a putative probiotic strain *Lactobacillus plantarum* EM isolated from kimchi. LWT‐Food Science and Technology, 62, 210–217. 10.1016/j.lwt.2015.01.019

[fsn31217-bib-0010] Choi, W. H. , Gwon, S. Y. , Ahn, J. , Jung, C. H. , & Ha, T. Y. (2013). Cooked rice prevents hyperlipidemia in hamsters fed a high‐fat/cholesterol diet by the regulation of the expression of hepatic genes involved in lipid metabolism. Nutrition Research, 33, 572–579. 10.1016/j.nutres.2013.04.006 23827132

[fsn31217-bib-0011] Folch, J. , Lees, M. , & Sloane Stanley, G. H. (1957). A simple method for the isolation and purification of total lipids from animal tissues. Journal of Biological Chemistry, 226, 497–509. 13428781

[fsn31217-bib-0012] Friedewald, W. T. , Levy, R. I. , & Fredrickson, D. S. (1972). Estimation of the concentration of low‐density lipoprotein cholesterol in plasma, without use of the preparative ultracentrifuge. Clinical Chemistry, 18, 499–502. 4337382

[fsn31217-bib-0013] Han, K.‐H. , Iijuka, M. , Shimada, K.‐I. , Sekikawa, M. , Kuramochi, K. , Ohba, K. , … Fukushima, M. (2005). Adzuki resistant starch lowered serum cholesterol and hepatic 3‐hydroxy‐3‐methylglutaryl‐CoA mRNA levels and increased hepatic LDL‐receptor and cholesterol 7α‐hydroxylase mRNA levels in rats fed a cholesterol diet. British Journal of Nutrition, 94, 902–908. 10.1079/bjn20051598 16351766

[fsn31217-bib-0014] Harb, A. A. , Bustanji, Y. K. , & Abdalla, S. S. (2018). Hypocholesterolemic effect of β‐caryophyllene in rats fed cholesterol and fat enriched diet. Journal of Clinical Biochemistry and Nutrition, 62, 230–237. 10.3164/jcbn.17-3 29892161 PMC5990408

[fsn31217-bib-0015] Hertog, M. , Feskens, E. , Kromhout, D. , Hertog, M. , Hollman, P. , Hertog, M. , & Katan, M. B. (1993). Dietary antioxidant flavonoids and risk of coronary heart disease: The Zutphen elderly study. Lancet, 342, 1007–1011. 10.1016/0140-6736(93)92876-U 8105262

[fsn31217-bib-0016] Jang, I. S. , Hwang, D. Y. , Lee, J. E. , Chae, K. R. , Kim, Y. K. , Kang, T. S. , … Cho, J. S. (2003). Physiological difference between dietary obesity‐susceptible and obesity‐resistant Sprague Dawley rats in response to moderate high fat diet. Experimental Animals, 52, 99–107. 10.1538/expanim.52.99 12806884

[fsn31217-bib-0017] Jeon, J. H. , Kim, B. , Mun, E. G. , Cha, Y. S. , & Yu, O. K. (2017). Effects of fermented blueberry liquid in high‐fat diet‐induced obese C57BL/6J mice. Journal of Nutrition and Health, 50, 543–551. 10.4163/jnh.2017.50.6.543

[fsn31217-bib-0018] Jeun, J. , Kim, S. , Cho, S.‐Y. , Jun, H.‐J. , Park, H.‐J. , Seo, J.‐G. , … Lee, S.‐J. (2010). Hypocholesterolemic effects of *Lactobacillus plantarum* KCTC3928 by increased bile acid excretion in C57BL/6 mice. Nutrition, 26, 321–330. 10.1016/j.nut.2009.04.011 19695834

[fsn31217-bib-0019] Jo, S. Y. , Choi, E. A. , Lee, J. J. , & Chang, H. C. (2015). Characterization of starter kimchi fermented with *Leuconostoc kimchii* GJ2 and its cholesterol‐lowering effects in rats fed a high‐fat and high‐cholesterol diet. Journal of the Science of Food and Agriculture, 95, 2750–2756. 10.1002/jsfa.7018 25425317

[fsn31217-bib-0020] Jung, J. H. , & Kim, H. S. (2013). The inhibitory effect of black soybean on hepatic cholesterol accumulation in high cholesterol and high fat diet‐induced non‐alcoholic fatty liver disease. Food and Chemical Toxicology, 60, 404–412. 10.1016/j.fct.2013.07.048 23900008

[fsn31217-bib-0021] Lee, J. , Kim, Y. , Yun, H. S. , Kim, J. G. , Oh, S. , & Kim, S. H. (2010). Genetic and proteomic analysis of factors affecting serum cholesterol reduction by *Lactobacillus acidophilus* A4. Applied and Environmental Microbiology, 76, 4829–4835. 10.1128/aem.02892-09 20495044 PMC2901756

[fsn31217-bib-0022] Lee, S. H. , & Chang, H. C. (2016). Isolation of antifungal activity of *Leuconostoc mesenteroides* TA from kimchi and characterization of its antifungal compounds. Food Science and Biotechnology, 25, 213–219. 10.1007/s10068-016-0032-8 30263260 PMC6049348

[fsn31217-bib-0023] Lee, S. G. , & Chang, H. C. (2018). Purification and characterization of mejucin, a new bacteriocin produced by *Bacillus subtilis* SN7. LWT ‐ Food Science and Technology, 87, 8–15. 10.1016/j.lwt.2017.08.044

[fsn31217-bib-0024] Leroy, F. , & De Vuyst, L. (2004). Lactic acid bacteria as functional starter cultures for the food fermentation industry. Trends in Food Science and Technology, 15, 67–78. 10.1016/j.tifs.2003.09.004

[fsn31217-bib-0025] Li, C. L. , Ding, Q. , Nie, S. P. , Zhnag, Y. S. , Xing, T. , & Xie, M. Y. (2014). Carrot juice fermented with *Lactobacillus plantarum* NCU116 ameliorates type 2 diabetes in rats. Journal of Agricultural and Food Chemistry, 62, 11884–11981. 10.1021/jf503681r 25341087

[fsn31217-bib-0026] Marazza, J. A. , LeBlanc, J. G. , de Giori, G. S. , & Garro, M. S. (2013). Soymilk fermented with *Lactobacillus rhamnosus* CRL981 ameliorates hyperglycemia, lipid profiles and increase antioxidant enzyme activities in diabetic mice. Journal of Functional Foods, 5, 1848–1853.

[fsn31217-bib-0027] Marco, M. L. , Heeney, D. , Binda, S. , Cifelli, C. J. , Cotter, P. D. , Foligné, B. , … Hutkins, R. (2017). Health benefits of fermented foods: Microbiota and beyond. Current Opinion in Biotechnology, 44, 94–102. 10.1016/j.copbio.2016.11.010 27998788

[fsn31217-bib-0028] Ministry of Health and Welfare (2013). Korea health statistics 2013: Korea national health and nutrition examination survey (KNHANES Ⅵ‐1) (pp. 659–668). Seoul, Korea: Sejong.

[fsn31217-bib-0029] Miyake, J. H. , Duong‐Polk, X. T. , Taylor, J. M. , Du, E. Z. , Castellani, L. W. , Lusis, A. J. , & Davis, R. A. (2002). Transgenic expression of cholesterol‐7‐α‐hydroxylase prevents atherosclerosis in C57BL/6J mice. Arteriosclerosis, Thrombosis, and Vascular Biology, 22, 121–126. 10.1161/hq0102.102588 https://doi.org/ 11788471

[fsn31217-bib-0030] Moon, S. H. , Kim, C. R. , & Chang, H. C. (2018). Heterofermentative lactic acid bacteria as a starter culture to control kimchi fermentation. LWT‐Food Science and Technology, 88, 181–188. 10.1016/j.lwt.2017.10.009

[fsn31217-bib-0031] Murakami, S. , Fujita, M. , Nakamura, M. , Sakono, M. , Nishizono, S. , Sato, M. , … Fukuda, N. (2016). Taurine ameliorates cholesterol metabolism by stimulating bile acid production in high‐cholesterol‐fed rats. Clinical and Experimental Pharmacology and Physiology, 43, 372–378. 10.1111/1440-1681.12534 26710098

[fsn31217-bib-0032] Nakamura, Y. , Kanazawa, M. , Liyanage, R. , Iijima, S. , Han, K.‐H. , Shimada, K.‐I. , … Fukushima, M. (2009). Effect of white wheat bread containing sugar beet fiber on serum lipids and hepatic mRNA in rats fed on a cholesterol‐free diet. Bioscience, Biotechnology, and Biochemistry, 73, 1280–1285. 10.1271/bbb.80787 19502741

[fsn31217-bib-0033] Ohm, I. Y. , Kim, S. Y. , & Jun, H. J. (2012). Report on Korean industry trends in fermented beverages (ed.). Sowon, Korea: Foundation of Agricultural Technology Commercialization and Transfer.

[fsn31217-bib-0034] Parvez, S. , Malik, K. A. , Ah Kang, S. , & Kim, H. Y. (2006). Probiotics and their fermented food products are beneficial for health. Journal of Applied Microbiology, 100, 1171–1185. 10.1111/j.1365-2672.2006.02963.x 16696665

[fsn31217-bib-0035] Reeves, P. G. , Nielsen, F. H. , & Fahey, G. C. (1993). AIN‐93 purified diets for laboratory rodents: Final report of the American institute of nutrition Ad Hoc writing committee on the reformulation of the AIN‐76A rodent diet. The Journal of Nutrition, 123, 1939–1951. 10.1093/jn/123.11.1939 8229312

[fsn31217-bib-0036] Rizvi, F. , Puri, A. , Bhatia, G. , Khanna, A. K. , Wulff, E. M. , Rastogi, A. K. , & Chander, R. (2003). Antidyslipidemic action of fenofibrate in dyslipidemic‐diabetic hamster model. Biochemical and Biophysical Research Communications, 305, 215–222. 10.1016/s0006-291x(03)00721-6 12745061

[fsn31217-bib-0037] Rosenfeld, L. (1989). Lipoprotein analysis. Early methods in the diagnosis of atherosclerosis. Archives of Pathology and Laboratory Medicine, 113, 1101–1110. 2679486

[fsn31217-bib-0038] Sun, F. , Xie, M. L. , Xue, J. , & Wang, H. B. (2010). Osthol regulates hepatic PPARα‐mediated lipogenic gene expression in alcoholic fatty liver murine. Phytomedicine, 17, 669–673. 10.1016/j.phymed.2009.10.021 20042322

[fsn31217-bib-0039] Wu, J.‐H. , Leung, G.‐H. , Kwan, Y.‐W. , Sham, T.‐T. , Tang, J.‐Y. , Wang, Y.‐H. , … Chan, S.‐W. (2013). Suppression of diet‐induced hypercholesterolaemia by saponins from *Panax notoginseng* in rats. Journal of Functional Foods, 5, 1159–1169. 10.1016/j.jff.2013.03.013

[fsn31217-bib-0040] Yang, E. J. , & Chang, H. C. (2010). Purification of a new antifungal compound produced by *Lactobacillus plantarum* AF1 isolated from kimchi. International Journal of Food Microbiology, 139, 56–63. 10.1016/j.ijfoodmicro.2010.02.012 20226553

[fsn31217-bib-0041] Yugarani, T. , Tan, B. K. , Teh, M. , & Das, N. P. (1992). Effects of polyphenolic natural products on the lipid profiles of rats diets. Lipids, 27, 181–186. 10.1007/BF02536175 1522762

